# A phylogenomic gene cluster resource: the Phylogenetically Inferred Groups (PhIGs) database

**DOI:** 10.1186/1471-2105-7-201

**Published:** 2006-04-11

**Authors:** Paramvir S Dehal, Jeffrey L Boore

**Affiliations:** 1 Evolutionary Genomics Department, DOE Joint Genome Institute and Lawrence, Berkeley National Laboratory, 2800 Mitchell Drive, Walnut Creek, CA 94598, USA; 2 Department of Integrative Biology, 3060 Valley Life Sciences Building, University of California, Berkeley, CA 94720, USA

## Abstract

**Background:**

We present here the PhIGs database, a phylogenomic resource for sequenced genomes. Although many methods exist for clustering gene families, very few attempt to create truly orthologous clusters sharing descent from a single ancestral gene across a range of evolutionary depths. Although these non-phylogenetic gene family clusters have been used broadly for gene annotation, errors are known to be introduced by the artifactual association of slowly evolving paralogs and lack of annotation for those more rapidly evolving. A full phylogenetic framework is necessary for accurate inference of function and for many studies that address pattern and mechanism of the evolution of the genome. The automated generation of evolutionary gene clusters, creation of gene trees, determination of orthology and paralogy relationships, and the correlation of this information with gene annotations, expression information, and genomic context is an important resource to the scientific community.

**Discussion:**

The PhIGs database currently contains 23 completely sequenced genomes of fungi and metazoans, containing 409,653 genes that have been grouped into 42,645 gene clusters. Each gene cluster is built such that the gene sequence distances are consistent with the known organismal relationships and in so doing, maximizing the likelihood for the clusters to represent truly orthologous genes. The PhIGs website contains tools that allow the study of genes within their phylogenetic framework through keyword searches on annotations, such as GO and InterPro assignments, and sequence similarity searches by BLAST and HMM. In addition to displaying the evolutionary relationships of the genes in each cluster, the website also allows users to view the relative physical positions of homologous genes in specified sets of genomes.

**Summary:**

Accurate analyses of genes and genomes can only be done within their full phylogenetic context. The PhIGs database and corresponding website  address this problem for the scientific community. Our goal is to expand the content as more genomes are sequenced and use this framework to incorporate more analyses.

## Background

The continually increasing number of whole genome sequencing projects has underscored the need for a high-throughput methodology to sort genes into orthologous sets to facilitate genome analysis. With a more robust understanding of the evolutionary history for each gene in the genome, not only can we more accurately transfer annotation across organisms, but we can also address larger biological questions regarding the evolution of genomes and species as well as the functional and biochemical processes encoded within each genome. Currently, most gene annotations rely on homologs identified by pair-wise sequence similarity to transfer the presumed function. This approach has been shown to have many drawbacks [1] which lead to annotation errors. Incorrect assignments are generally due to gene duplication events [2] giving rise to paralogs that can then acquire a new function or sub-functionalize [3,4], accelerated rates of amino acid substitution [5] and domain shuffling [6]. Simple pair-wise comparisons cannot uncover these events.

Several approaches have been proposed to address these problems. However, most of these retain the problems associated with simply clustering genes based on sequence similarity and fail to incorporate the known evolutionary relationships of species [7-9]. Alternatively, those approaches that attempt to use some aspect of the evolutionary relationships of the species to inform the clustering process fail to then create a phylogenetic tree to uncover the relationships of the genes within the clusters [10-12].

The method we present here considers *a priori *the known evolutionary relationships among the considered organisms as a guide to constructing gene clusters, then analyzes each cluster for the evolutionary relationships among the contained genes in order to reconstruct the evolutionary history of each gene family using standard analytical methods of molecular evolution. This provides a tool for the scientific community for gaining a more complete understanding of such things as evolutionary patterns of gene duplication and loss, variation in rates of amino acid substitution, and alterations in gene structure. PhIGs is the first truly comprehensive whole genome analysis phylogenetic tool allowing for accurate assessment of gene family and genome structure evolution.

## Construction and content

In this work, we develop a computational framework for the identification of sets of genes which have all descended from a single ancestral gene in the common ancestor of the lineages being examined. This collection of genes is then followed by the construction of phylogenetic trees for each set to determine relationships of the gene cluster members.

A relational database is used to store the genome annotations for each taxon. All sequence data as well as individual gene annotations, including InterPro [13] and Gene Ontology [14] assignments, intron, exon and UTR structural information, and genomic positional information are retrieved whenever available. In addition, results of analyses such as sequence alignments, intermediate data, and trees are stored in the database. Table 1 lists the genomes included in the current data set, which will be updated as more genome sequences become available.

**Figure 1:**
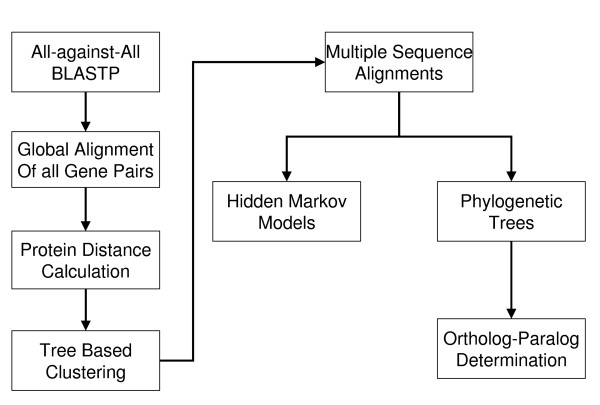
**Flowchart of the PhIGs process**. This is a graphical overview of the pipeline for processing gene models from many genomes into the PhIGs analysis.

The overall process involves five stages (Figure 1) explained in more detail below: (1) an all against all BLASTP [15] of the complete proteomes; (2) global alignment and distance calculation of the gene pairs identified by BLAST; (3) iterative, hierarchical clustering; (4) multiple sequence alignment (MSA) creation and editing; and (5) gene tree reconstruction.

### All against all BLASTP and global alignment

An all-against-all BLASTP search is performed on the entire protein dataset derived from each genome. Because each BLAST only reports local alignments, a global alignment is created for each protein pair returned by BLAST with ClustalW [16]. A protein distance is then calculated using the JTT matrix and the protdist program from PHYLIP [17], hereafter referred to as the distance between genes themselves. These pair-wise protein distances and gap-free alignment lengths are then used as input for the clustering process. All alignments are stored in the PhIGs database.

### Gene clustering

**Figure 2:**
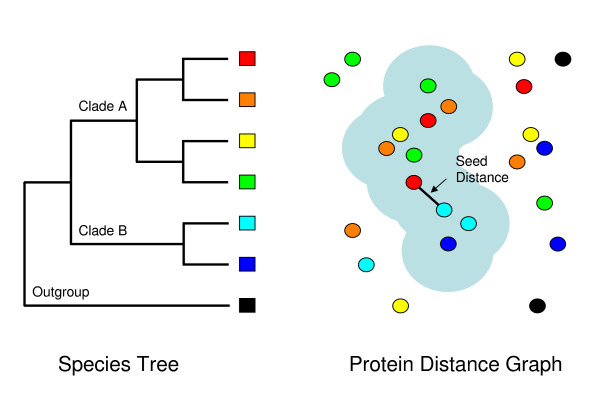
**Illustration of the clustering method**. The tree shown on the left side of the figure indicates the evolutionary relationships among several hypothetical organisms, four from Clade A, two from Clade B, and one that is an outgroup. The right side of the figure illustrates a protein distance graph with circles representing proteins colored to conform to each organism, with the spatial distance of the circles proportional to their sequence distance. The cluster is created by identifying a pair of sequences (a seed) that is the shortest distance from any Clade A protein to any Clade B protein. The cluster is then grown by adding all proteins that have a shorter distance than the seed until no additions can be made. The blue cloud represents one such cluster. See text for more details.

Gene clustering is performed at each node of the tree, using the known evolutionary relationships of the organisms and all pair-wise protein distances as input. The objective of the clustering process is to create gene clusters at each node of the evolutionary tree such that the genes of the descending taxa are more closely related to each other than they are to the genes from the outgroup taxa. We employ a hierarchical approach, starting at the base of the best known evolutionary tree of the organisms, and proceeding up the tree iteratively. For each bifurcating node, taxa are temporarily grouped such that those on one descending branch are labeled as clade A and those on the other as clade B. The remaining taxa, having branched earlier, are considered to be the outgroup (Figure 2). Clusters of genes are then constructed such that the included genes meet the following criteria: (1) Genes from organisms within clade A are more similar to each other than they are to genes from organisms within clade B; and (2) genes from clade A and clade B are more closely related to each other than they are to any gene in the outgroup. Effectively, this can be achieved by first finding the top scoring alignment for each gene within any member of its sister clade, then recruiting all additional genes that have greater similarity to either one of these genes using single linkage clustering with inclusion criteria being set to the distance and alignment length of the alignment of the seed. As illustrated in Figure 2, the initial seed alignment of a pair of genes, one from each of clade A and clade B, defines an area shown in blue around representing the minimum match quality. As more genes are added to the cluster, this area grows until no more genes can be added.

Because this clustering approach is dependent on seeds, the order in which the seeds are processed will affect the clustering results. To ensure that each gene is placed in its optimal cluster, a greedy approach is used by sorting the list of seed alignments by the BLASTP score and processing the seeds by using the highest scoring seed first. In so doing, any subsequent cluster that attempts to incorporate a gene which has already been clustered can be eliminated. It is important to note that the BLASTP score is only used to sort the seeds and clustering is based on the protein distance and alignment length. The pseudocode describing this method is available online as additional file 1: Cluster Pseudocode.

By using an iterative approach, working through the entire evolutionary tree of the organisms beginning at the base, we ensure that the most early diverging gene families create the most comprehensive clusters, with later established families properly assigned to the lineages in which they arose. Genes with a highly accelerated amino acid substitution rate, such that they are more distantly related to their sister genes than those sister genes are to a gene from the outgroup, are always excluded, since this cannot be differentiated from ancestral paralogy.

### MSA and phylogenetic tree creation

A multiple sequence alignment (MSA) is created for each cluster using the ClustalW [16] program, which provides the input for phylogenetic tree reconstruction. Alignments are trimmed to remove columns that contain gap characters and the cluster is eliminated if the resulting alignment contains fewer than 100 aligned amino acid positions. Phylogenetic trees are created using the quartet puzzling maximum likelihood method implemented in the TREE-PUZZLE [18] program using the JTT model of amino acid substitution and a gamma distribution of rates over eight rate categories with 10,000 puzzling steps to assess reliability. Quartet puzzling is chosen here as a compromise between speed and reliability; however, the multiple sequence alignment is available for re-analysis with other tree reconstruction methods. The resulting gene tree is then reconciled with the known relationships of the organisms to determine, relative to lineage splitting, when each duplication or loss occurred, and so to determine an initial estimate of the orthology and paralogy relationships among the genes. The reconciliation process uses the most straight-forward interpretation of the tree; no alterations are made to minimize the number of duplications or gene loss events. Genes are considered orthologs if they are separated only by speciation nodes consistent with the known phylogenetic tree and considered paralogs if there is a node representing a duplication event in their shared ancestry.

The MSAs are also used to create Hidden Markov Models (HMMs) to later facilitate searching the clusters and to provide a resource for placing genes from genomes too sparsely sampled to be included in this comprehensive analysis, such as those from many EST sequencing projects.

An instructive example of this process is for the Succinyl-Coenzyme A ligase beta subunit family. In this example, considering the fungi and metazoans first as clade A and clade B, respectively, the seed alignment used is the match between a gene from *M. grisea *and one from mouse (Sucla2). The protein distance measure and gap free alignment length of this seed alignment pair is now taken to represent the maximum distance and minimum alignment length for recruiting new genes to the cluster. Any fungal or metazoan gene with a shorter distance and larger alignment length is added. In this case the fungal gene recruits a single gene from each of the remaining fungal genomes and the mouse gene recruits two genes in each case from most of the remaining metazoan genomes and three genes from each of human, chimp, and mouse. All of these genes now included in the cluster have matches to each other that are as good or better than the initial seed alignment and do not have better matches to any other cluster. The phylogenetic tree created for this cluster ultimately shows that this gene family had a duplication at the base of the metazoan lineage, another duplication at the base of the primate lineage, and an independent duplication in the mouse lineage.

## Utility

### Cluster view

**Figure 3:**
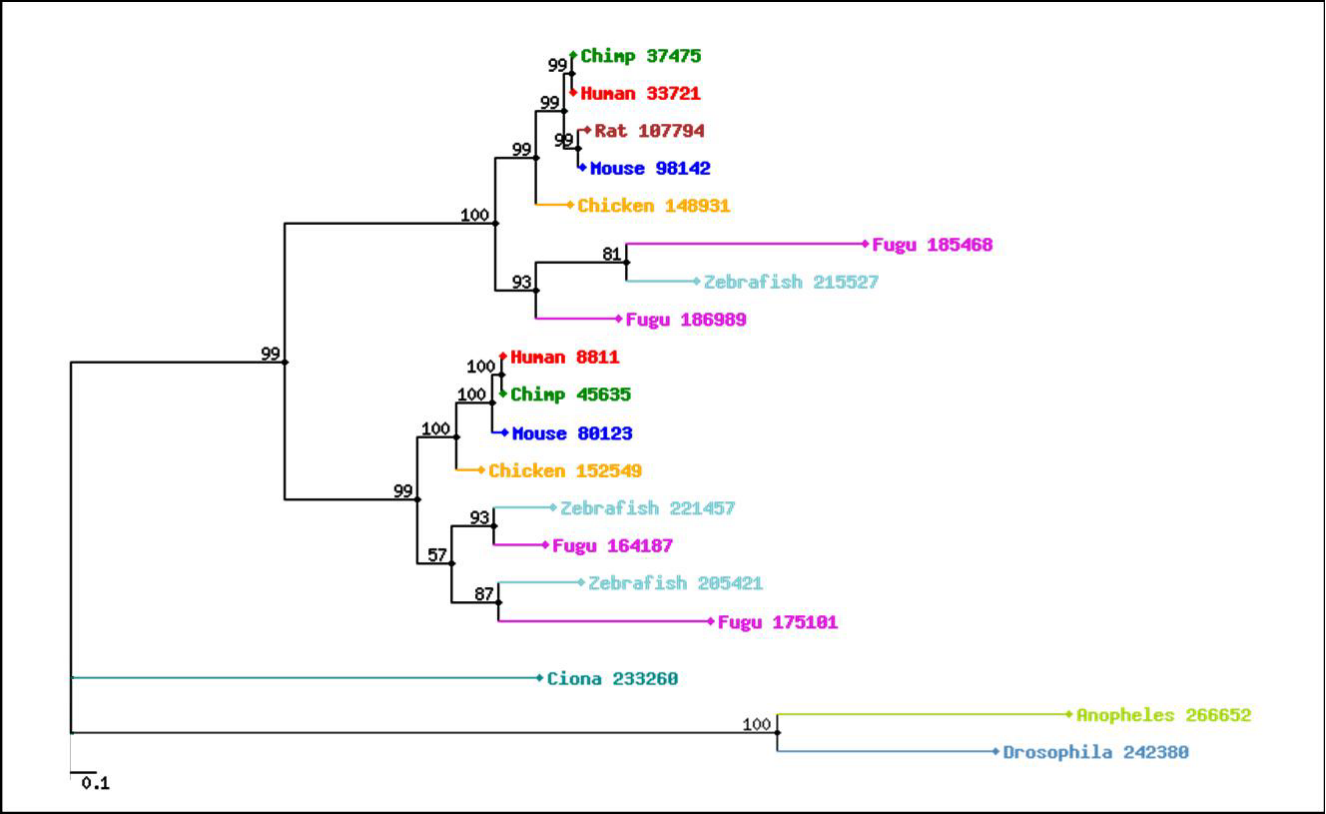
**An example phylogenetic tree**. This is one output of the PhIGs analysis that is shown on the Cluster View webpage. Instead of simply listing the members of a cluster, a phylogenetic tree is created showing the evolutionary relationships of this multigene family. In this example, we can see that this family had gene duplication events at the base of vertebrates and in the fish lineage. Because the branch lengths are proportional to the rate of amino acid substitutions, we can see how rates of evolution have varied.

The PhIGs database allows users to view genes within the evolutionary context of other sequenced genomes. Because each cluster is constructed to represent the extant descendants of a single ancestral gene, the gene trees provided allow the user to see where gene duplication events have occurred and the rates of amino acid sequence change along the individual branches of the tree (Figure 3). By reconciling the gene tree with the species tree, orthology and paralogy relationships can be determined.

Comparisons of differences and similarities in annotations, such as definition line (defline) gene descriptions, InterPro families, and Gene Ontology assignments, can be made with respect to the tree. The user can make a determination of whether the gene annotations are consistent with the tree topology and whether annotations should be transferred to unannotated genes. Additionally, the genomic location and intron and exon structure of each gene is also provided, enabling analysis of such issues as whether the paralogous genes are physically clustered within a genome, indicating tandem or segmental duplication, or whether the gene family is widely dispersed. Alterations in gene intron and exon structure (and sizes) relative to other members of the cluster may be the result of biological forces acting on the genome or may simply be indicative of poor gene modeling.

The MSA for each cluster is also made available in the Cluster View. An alignment graphic, with the intron and exon structure superimposed, is shown on the page and a detailed alignment view is provided through a Jalview [19] java applet. By examining the MSA, the user can determine whether poorly aligning or missing regions of a gene contains a protein domain which may indicate the gain or loss of some function. Of course, when dealing with gene models of unknown quality, the genomic sequence should be examined for the possibility of annotation error before concluding an exon or domain loss occurred.

### Gene view

All annotations related to each gene are viewable on its Gene View web page. This includes the annotations presented on the Cluster View page as well as a summary of domains found with the InterProScan [20] program (not available for all genomes) and a summary of all pair-wise alignments, including the calculated protein distance. This pair-wise alignment information can be useful to determine whether any genes may have been left out of the cluster for failing to meet the distance and alignment length cutoffs. In some cases, this appears to be a gene model that is erroneously fragmented or merged with another, and so PhIGs provides a powerful tool for detecting these potential errors.

### Searching

Searches of the database can be done by sequence similarity or by text matches to annotation fields. Text searches can be done on gene names, deflines, or InterPro annotations. Because these are associated with individual genes, the search function can be used to either return a list of genes from a selected set of taxa that contain the search term or it can return a set of clusters which contain genes matching the search term. Because all clustering is done at the protein level, sequence similarity searches can only be performed against protein datasets. An individual sequence can be aligned against the proteins contained in the database using the BLAST program. Matches to the sequence can then be used as an entry into the cluster in which they belong. Alternatively, a similarity search can be performed directly against the Hidden Markov Models (HMMs) generated from the MSA of the clusters using the HMMER [21] program. Once a match has been made, the user can easily download either the raw fasta file of the cluster or the MSA file to create a tree incorporating the new sequence.

### Synteny maps

**Figure 4:**
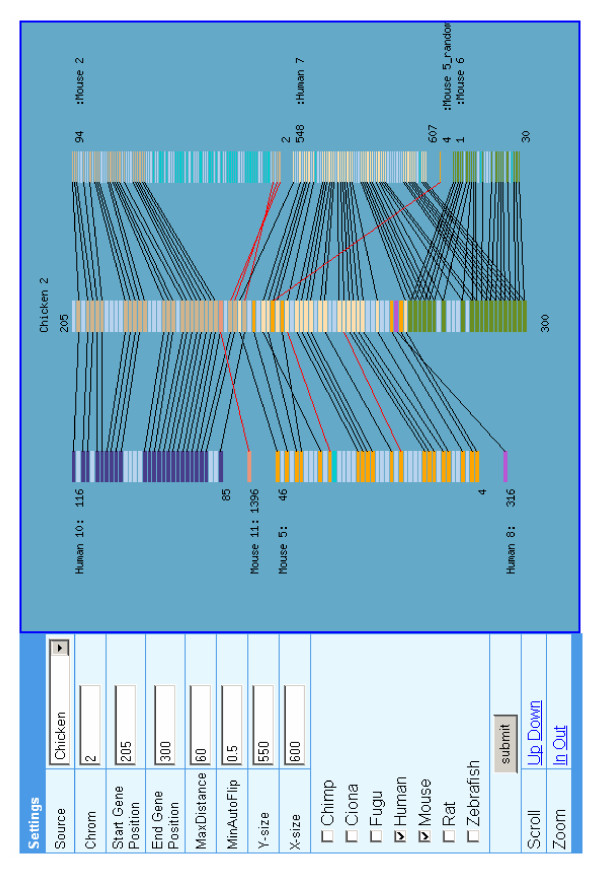
**An example Synteny Map**. Genes ranging from number 205 through 301 on chicken chromosome 2 (numbered as they occur from the p-telomere to q-telomere along the chromosome) are shown as rectangles in the center of the diagram. On the left and right are the orthologs of these genes found in the human and mouse genomes as determined by the PhIGs analysis, shown as they are arranged. Black connecting lines join orthologs in the same relative transcriptional orientation whereas red lines indicate those that are inverted. Blue rectangles indicate intervening genes without identified orthologs in the genomes being compared. Cyan rectangles that do not have connecting lines, as can be seen for a portion of mouse chromosome 2, indicate that orthologs exist in chicken (the query genome), but not in the portion specified for this page.

These analyses produce sets of true, one-to-one orthologs, and this presentation incorporates a view of their relative physical positions across multiple genomes. As opposed to other methods that rely on sequence similarity to create comparative genome alignments, this avoids confusion that arises from paralogy. Synteny maps are generated by selecting a genomic span from a single reference genome and one or more query genomes to align (Figure 4). All identified orthologous genes between the selected genome and each of the query genomes are shown.

## Discussion and conclusion

The rapidly increasing number of sequenced genomes allows us to study genes and genomes within an evolutionary context. Not only does this assist in the transfer of annotations between genes, but also allows us to uncover how the forces of evolution have shaped each genome. The PhIGs database project seeks to facilitate comparative genomic, phylogenomic, and functional genomic studies by providing a comprehensive resource for the determination of the evolutionary history for all genes from the fully sequenced genome projects. The two main properties that differentiate the PhIGs database from other clustering methods are the use of the known evolutionary relationships of the species to create gene clusters representing the descendants of a single ancestral gene and the creation of a complete phylogenetic gene tree of the cluster members using widely accepted analytic methods of molecular evolution. By combining this phylogenetic information with functional annotation, gene structure, genomic position and other datasets, the PhIGs database will prove to be a valuable resource for all fields of biology currently using genomic data.

The scientific applications of the PhIGs database are broad, extending beyond practical genome annotation and analysis. For instance, obvious applications are the use of orthogous gene clusters for: (1) organismal phylogenetic reconstruction; (2) the study of genome evolution by gene duplication; (3) gene structure evolution through the gain and loss of exons, introns, and domains; (4) the identification of gene family expansions and losses and 5) genome evolution. The PhIGs analyses have already been used to compare specifically the whole genomes of a tunicate, fish, mouse, and human, demonstrating that the relative positions in the human genome of paralogs generated by duplications at the base of vertebrates provide clear evidence in favor of the contentious hypothesis of two rounds of whole genome duplication having occurred at the base of the vertebrates, and perhaps providing the raw material for vertebrate complexity [22]. Further applications can be developed to meet other analytical needs of the scientific community.

Future development includes improvements to the underlying clustering method, incorporation of more annotation data, creation of more analysis tools and more rapid updates of newly available genomes. The functionality of the PhIGs database is currently accessible though the web interface and data files of orthology relationships for download. Our goal is to convert this into an open source project to help maintain and expand this as a resource for the scientific community.

## Authors' contributions

Both authors conceived and designed the project. PSD did all of the programming and implementation. Both authors wrote and approved the final manuscript.

## Supplementary Material

Additional File 1 Pseudocode. The pseudocode for PhIGs database construction Click here for file

## Figures and Tables

**Table 1:** Taxa currently included in PhIGs

**Species**	**Taxonomy**	**Genome source**	**URL**
*Homo sapiens*	Primates	Ensembl 22.34d.1	
*Pan troglodytes*	Primates	Ensembl 22.1.1	
*Mus musculus*	Rodentia	Ensembl 22.32b.1	
*Rattus norvegicus*	Rodentia	Ensembl 22.3b.1	
*Gallus gallus*	Aves	Ensembl 22.1.1	
*Takifugu rubripes*	Pisces	Ensembl 22.2c.1	
*Danio rerio*	Pisces	Ensembl 22.3b.1	
*Ciona intestinalis*	Urochordata	JGI ciona4	
*Drosophila melanogaster*	Arthropoda	Ensembl 22.3a.1	
*Anopheles gambiae*	Arthropoda	Ensembl 22.2b.1	
*Caenorhabditis elegans*	Nematoda	Ensembl 22.116a.1	
*Caenorhabditis briggsae*	Nematoda	Ensembl 24.24.1	
*Ustilago maydis*	Basidiomycota	Broad release 2	
*Phanerochaete chrysosporium*	Basidiomycota	JGI whiterot1	
*Cryptococcus neoformans*	Basidiomycota	TIGR CNA1	
*Schizosaccharomyces pombe*	Ascomycota	Pompep version 19	
*Aspergillus nidulans*	Ascomycota	Broad release 1.3	
*Fusarium graminearum*	Ascomycota	Broad release 1.1	
*Trichoderma reesei*	Ascomycota	JGI trire1	
*Magnaporthe grisea*	Ascomycota	Broad release 2.4	
*Neurospora crassa*	Ascomycota	Broad release 3	
*Candida albicans*	Ascomycota	Stanford release 19	
*Saccharomyces cerevisiae*	Ascomycota	Saccharomyces Genome Database	
