# Supplementary material for: A phylogenomic gene cluster resource: the Phylogenetically Inferred Groups (PhIGs) database
Source: BMC Bioinformatics. 2006 Apr 11;7:201. doi: 10.1186/1471-2105-7-201 (PMC1523372; doi:10.1186/1471-2105-7-201)
Supplement: Additional File 1 — Pseudocode. The pseudocode for PhIGs database construction [file 1471-2105-7-201-S1.pdf]

```
//create protein distance graph
graph G = (v,e)

For each gene i ∈ {all genes}
    Vertex v1 := ( i )
    G.ADD_VERTEX( v1 ) if v1 ∉ {VERTICES (G)}
    For each gene j ∈ {all genes}
        Vertex v2 := ( j )
        G.ADD_VERTEX if v2 ∉ {VERTICES (G)}
        Alignment := BLASTP.BestHit( i, j )
        Next if | Alignment | == 0
        Edge e := (v1, v2 )
        e.score           = Alignment.score
        e.alignmentLength = ClustalW gap-free alignment length
        e.distance        = protdist distance
        G.ADD_EDGE(e)

tree T := known evolutionary tree of taxa
For each node ∈ {T}
    Next if | node.children | ≠ 2
    Outgroup := {taxa ∉ node.childrenTaxa}

    //create seeds
    Seeds := {}
    For each gene i ∈ {node[0]}
        Seeds[i] := MAX { G[i][x].score } where x ∈ {node[1].genes}
    For each gene i ∈ {node[1]}
        Seeds[i] := MAX { G[i][x].score } where x ∈ {node[0].genes}

    SORT Seeds descending

    //create clusters
    geneUsed := {}
    For each seed ∈ {Seeds}
        minDistance := seed.distance
        minAlignmentLength := seed.alignmentLength

        //depth first search
        Cluster := {genes ∈ {seed}}
        Seen    := {}
        For each gene i ∈ {Cluster}
            Next If Seen[i] == TRUE
            For each gene j ∈ {G[i]}
                If G[i][j].distance < maxDistance
                  AND G[i][j].alignmentLenth > minAlignmentLength
                  AND Seen[j] == FALSE
                     ADD j to Cluster
                     Seen[j] := TRUE

    For each gene i ∈ {Cluster}
        If geneUsed.i == TRUE OR i.taxa ∈ {Outgroup}
            deleteCluster := TRUE
        geneUsed.i := TRUE

    If deleteCluster == TRUE
        Delete Cluster
    Else
        Return Cluster
```
